# Jurassic scorpionflies (Mecoptera) with swollen first metatarsal segments suggesting sexual dimorphism

**DOI:** 10.1186/s12862-021-01771-3

**Published:** 2021-03-20

**Authors:** Yan-jie Zhang, Peter J. M. Shih, Jun-you Wang, Maria E. McNamara, Chungkun Shih, Dong Ren, Tai-ping Gao

**Affiliations:** 1 College of Life Sciences and Academy for Multidisciplinary Studies, Capital Normal University, 105 Xisanhuanbeilu, Haidian District, Beijing, 100048 China; 2 Academy for Allied Health Sciences, 1776 Raritan Road, Scotch Plains, NJ 07076 USA; 3 Inner Mongolia Museum of Natural History, No.13, South 2nd Ring Road, Saihan District, Hohhot City, 010010 Inner Mongolia China; 4 School of Biological, Earth and Environmental Sciences, University College Cork, Cork, T23 TK30 Ireland; 5 Department of Paleobiology, National Museum of Natural History, Smithsonian Institution, Washington, DC 20013-7012 USA

**Keywords:** Fossil insect, Holcorpidae, Mesozoic, Orthophlebiidae, Tarsal swelling, Nuptial gift, Yanliao Biota

## Abstract

**Background:**

Sexual dimorphism is widespread in insects. The certain specialized structures may be used as weapons in male–male combats or as ornaments to enhance mating opportunities.

**Results:**

We report striking swollen first tarsal segments in two families, four genera and six species of scorpionflies from the Middle Jurassic Yanliao Biota of Northeastern China. Swollen tarsal segments are restricted to male specimens and to hind leg tarsi. The geometric morphometric analyses reveal that the degree of swelling within the orthophlebiid species possessing swollen first metatarsal segments is species-specific, which can be used as a diagnostic character for taxonomic and phylogenetic studies.

**Conclusions:**

The new findings indicate that swollen first metatarsal segments are relatively common in the family Orthophlebiidae during the Middle Jurassic. The tarsal swellings are considered to be sexually dimorphic, potentially associated with sexually display by males and/or camouflage of a “nuptial gift” in the mating process.

**Supplementary Information:**

The online version contains supplementary material available at 10.1186/s12862-021-01771-3.

## Background

Specializations of insect legs are relatively universal and potentially multifunctional for many insects, such as feeding, predation, fighting, digging, jumping, swimming, walking on water, etc. [1]. Modifications of the tarsi are known in extant insects. For example, webspinners (Embioptera) and the flies of the genus *Hilara* (Empididae, Diptera) produce silk from enlarged prothoracic basitarsi [2–4]. Certain bee species use their strigilis, combs of the spurs on the first tarsal joints of their forelegs, for cleaning antennae [5]. Oversized tarsi in male dance flies (*Empis* sp. (Empididae, Diptera)) are a secondary sexual character [6]. Male carabid beetles (Coleoptera) have broadened tarsi; Cerambycidae and Scarabaeidae (Euchirinae and Cetoniinae) have prolonged tarsi. Many representatives of beetles possess specialised setae on the tarsi, presumably enabling them to grasp to female elytra during copulation; male diving beetles, most notably Dytiscidae, possess adhesive structures on tarsi [7–9].

Similar tarsal modifications are rarely found in fossil insects. The only examples known are preserved in male specimens of the scorpionflies, *Orthophlebia elenae* and *Orthophlebia longicuada* from the Upper Jurassic Karabastau Formation in Russia, which have swollen first tarsal segments in the hindlimbs. These structures were originally interpreted as a “metatarsal organ” of unknown function [10, 11].

Here, we report the preservation of similar enlarged metatarsal segments in abundant scorpionflies from the Middle Jurassic (late Callovian, ca. 165–164 Mya) [12–15] Jiulongshan Formation at Daohugou, Inner Mongolia, China. Most of the studied specimens belong to the Orthophlebiidae; one specimen is a newly collected holcorpid species, *Conicholcorpa longa* sp. nov. We tested whether the tarsal morphology is sexually dimorphic, providing new insights into the behaviour and life history of Jurassic scorpionflies.

## Results

We studied and reported a total of 87 scorpionfly specimens, including one new holcorpid (*Conicholcorpa stigmosa* sp. nov.) and 86 orthophlebiids in five known species. The orthophlebiids represent five species belonging to three genera (Additional file 1: Figures S1–S5): *Orthophlebia extensa* Martynov, 1937; *Orthophlebia elenae* Willmann & Novokshonov, 1998; *Mesopanorpa densa* Zhang, 1996; *Mesopanorpa luanpingensis*, Hong, 1983; *Juraphlebia eugeniae* Soszyńska-Maj and Krzemińsk, 2020.

### Systematic palaeontology

Order Mecoptera Packard, 1886.

Family Holcorpidae Willmann, 1989.

Genus *Conicholcorpa* Li, Shih, Wang & Ren, 2017.

Type species *Conicholcorpa stigmosa* Li, Shih, Wang & Ren, 2017.

*Conicholcorpa longa* Zhang, Shih & Ren sp. nov.

**Holotype.** CNU-MEC-NN2015108p/c, male, part and counterpart. Measurements (length in mm) of the holotype are: body excluding antenna, 50.0; forewing, 20.0; hind wing, 16.7; the 6th, 7th, 8th segment of abdomen (A6, A7, A8) 9.2, 13.3 and 14.8, respectively.

**Characterization.** Mid-sized insect with the 6th, 7th and 8th segments of abdomen extremely elongated. The 6th segment of abdomen equal to the combination of head, thorax, and 1st to 5th segments of abdomen. The abdominal segment A7 is nearly 1.5 times as long as A6, just slightly shorter than A8. Forewing: Rs with six branches, M forking almost at the same level or slightly distal to Rs forking, several transverse veins dispersed among Rs.

**Remark. ***Conicholcorpa longa* sp. nov. is placed in *Conicholcorpa* Li, Shih, Wang & Ren, 2017 based on its forewing with M forking almost at the same level of Rs, the 7th segment (A7) much longer than 6th segment (A6) of the abdomen, the 6th segment (A6) without spur at the posterior margin, and A6, A7 and the 8th segment (A8) all straight. *Conicholcorpa longa* sp. nov. differs from the type species *Conicholcorpa stigmosa* (male) by the length of A6 nearly equal to the length of anterior part of the body before A6 (excluding antenna), A6 much thicker than A7 or A8, and A8 just slightly longer than A7.

**Etymology.** The specific name is derived from the Latin word ‘longus’, indicating the elongated and extended terminalia of this taxon.

**Type locality.** Collected from the Jiulongshan Formation of Daohugou Village, Shantou Township, Ningcheng County, Inner Mongolia, China, latest Middle Jurassic, as for all new orthophlebiid specimens in this study.

**Fig. 1:**
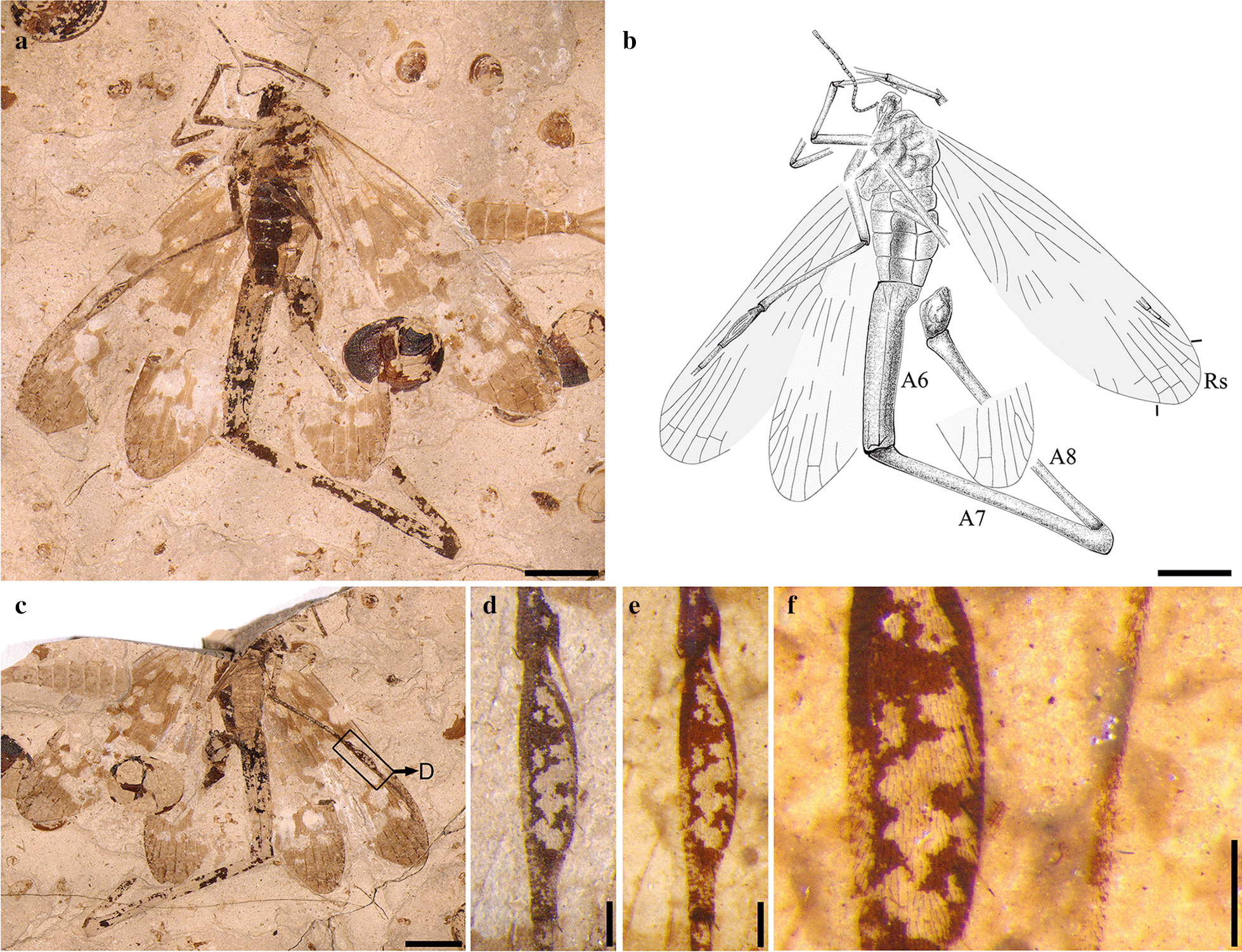
Photographs and line drawing of the holotype of *Conicholcorpa longa* sp. nov. **a**,** b** Habitus and line drawing of part for CNU-MEC-NN2015108p. **c** Habitus of counterpart for CNU-MEC-NN2015108c. **d** Swollen first segment of the metatarsus of (**c**). **e** Under ethanol. **f** Enlarged part of (**e**). Scale bars represent: 4 mm in (**a**–**c**); 0.5 mm in (**d**–**f**). A6–A8: the sixth to eighth abdominal segments

**Description.** Head: subcircular, relatively small; antenna filiform, at least 23 flagellomeres; mouthpart elongated (Fig. 1a, c).

Thorax: prothorax, mesothorax and metathorax discernible; forelegs: tibia (3.04 mm) slightly longer than the basitarsus (2.17 mm) and bears two spurs at apical; basitarsus as long as the following three segments combined; third tarsal segment shortest; the mid legs disarticulated and not preserved; tibia of hind legs (6.64 mm) much longer than the basitarsus (3.00 mm), two long spurs also fixed on the top of tibia; the 1st segment of metatarsus, spindle-shaped, the widest point about 0.70 mm in diameter (Fig. 1d, e); the swollen segments covered with dense setae and several setae much longer than others (Fig. 1f).

Wings: forewing with R forking at one-fourth of wing length; the branches of M not obvious, but the points of forking very close; Rs with 6 branches, M with 5 branches; hind wing not clear, due to poor preservation.

Abdomen: segments clear, the lengths of A1–A5 segments nearly equal and the third segment (A3) widest; A6–A8 elongated (Fig. 1b), A6 without two tergal spurs, A8 distinctly longest; A6 with rough surface, slightly tapering toward the terminal; A7 and A8 enlarged and slender, clearly thickened at both ends.

Genitalia: male external genitalia relatively large compared with the terminal of A8; genital bulb enlarged; dististyli not preserved, details invisible.

Family Orthophlebiidae Handlirsch, 1906;

Genus *Orthophlebia* Westwood, 1845.

*Orthophlebia extensa* Martynov, 1937.

Description of new materials: Head roughly circular, antenna filiform, chewing mouthparts robust and long, compound eye oval and large. Pronotum short, 0.6 times as long as mesonotum; metanotum 1.2 times as long as mesonotum. Forewing: long and relatively broad (elliptical to spatulate) with several anomalous white spots; Sc long, reaching the costal margin with a crossvein to C; R long, unforking and curving just before reaching the wing margin; the stem of Rs 1.8 times as long as that of Rs + MA while the stem of MA 1.6 times as long as the stem of Rs + MA; Rs with five branches, MA two branches, MP five branches; CuA long, connecting to MP with a short crossvein; CuP fusing with CuA basally. Hind wing similar to forewing but smaller, Sc short, reaching nearly the middle of the costal margin, MP four branches. Abdomen with nine segments, the last segment of male enlarged, bulbous, resembling the stinger of a scorpion (Additional file 1: Figure S1).

*Orthophlebia elenae* Willmann & Novokshonov,1998.

Description of new materials: Forewing: Sc short and straight without a crossvein to C, and ending at anterior margin slightly distal of the middle of the wing; R long, unforking and curving just before reaching the wing margin; the stem of Rs + MA almost as long as that of Rs, the forking of Rs distal of the forking of MA, Rs with four branches, MA two branches, MP five branches, forking distal of the forking of the stem of Rs + MA, CuA connecting to MP with a short crossvein; CuP fusing with CuA basally, A1, A2, A3 veins long and parallel to each other. Hind wing, MP with four branches; two crossveins between A1 and A2, one crossvein between A2 and A3 (Additional file 1: Figure S2).

Genus *Mesopanorpa* Handlirsch, 1906.

*Mesopanorpa densa* Zhang, 1996.

Description of new materials: Forewing: long and relatively broad (elliptical to spatulate); Sc long, reaching the costal margin with a crossvein to C; R long and straight, parallel to Sc and ending at anterior margin just distal of ending of Sc; the stem of Rs + MA forking at one-third of wing length from base; Rs with five branches, MA two branches, MP five branches; stem of Rs two times as long as that of MA and equal to stem of Rs + MA in length; stem of R + Rs + MA almost as long as that of Rs + MA; MP, forking slightly distal of the forking of Rs + MA, with five branches, CuA long, curving slightly, connecting to MP near base by a short crossvein; A1, A2 and A3 veins long and straight (Additional file 1: Figure S3).

*Mesopanorpa luanpingensis* Hong, 1983.

Description of new materials: Forewing: long and relatively broad (elliptical to spatulate); Sc long, almost parallel with C with a crossvein to C; the stem of R + Rs + MA bending posteriorly at one-fourth of wing length, R parallel to Sc and ending at anterior margin just distal of ending of Sc; the stem of Rs + MA forking at one-third of wing length from base; Rs with four branches, MA two branches, MP five branches; stem of Rs 1.5 times as long as that of MA and equal to that of Rs + MA in length; stem of R + Rs + MA about two times as long as that of Rs + MA; MP, forking just distal of the forking of Rs + MA, with five branches, CuA long, curving slightly, connecting to MP near base by a short crossvein; A1, A2 veins long and straight while A3 curving (Additional file 1: Figure S4).

Genus *Juraphlebia* Soszyńska-Maj & Krzemińsk, 2020.

*Juraphlebia eugeniae* Soszyńska-Maj & Krzemińsk, 2020.

Description of new materials: Forewing: long and relatively broad (elliptical to spatulate), with a large area of color marking at base, middle and distal part of the wing; Sc long, reaching the pterostigmal area with a crossvein to C; R long, unforking and curving just in the pterostigmal area, thickened and convex dorsally; the stem of R + Rs + MA 0.9 times as long as stem of Rs + MA, Rs with five branches, MA two branches, Rs and MA forking almost at the same level; MP with five branches, Rs forking slightly before MP; CuA connecting to MP with a short crossvein; CuP fusing with CuA basally; A1, A2, A3 veins long, reaching the anal margin. Hind wing, narrower than forewing, shaped nearly triangular; Sc short without crossvein to C, MP with four branches (Additional file 1: Figure S5).

### Different degree of metatarsal swellings of scorpionflies

**Fig. 2:**
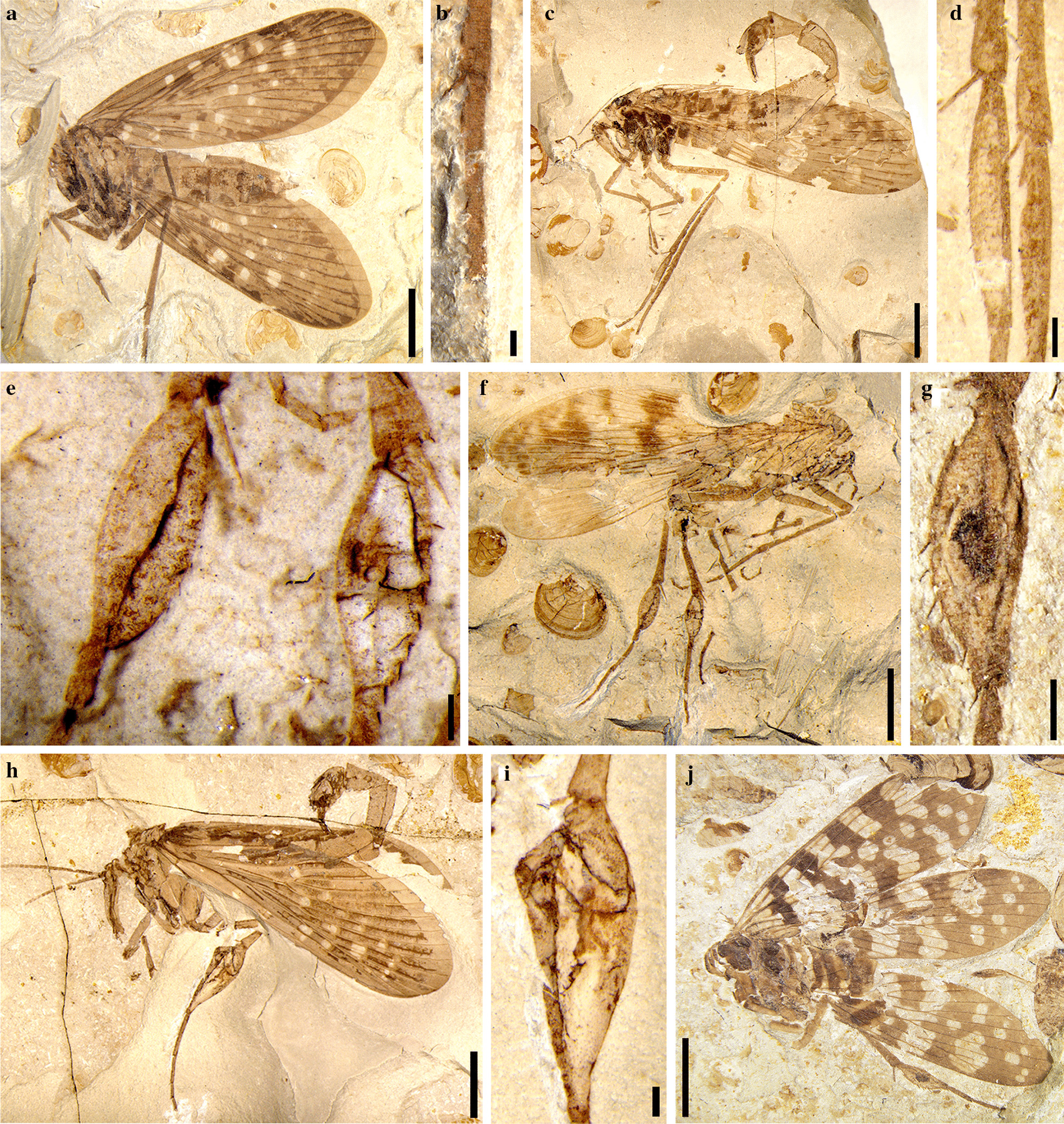
Comparison of non-swollen versus swollen first metatarsal segments. **a**, **b** Non-swollen, *Orthophlebia extensa* (CNU-MEC-NN2014059). **c**, **d** Slightly swollen, *Orthophlebia elenae* (CNU-MEC-NN2014005). **e**, **f** Moderately swollen, *Juraphlebia eugeniae* (CNU-MEC-NN2014009). **g**, **j** Heavily swollen, *Mesopanorpa luanpingensis* (CNU-MEC-NN2016222). **h**, **i** Extremely swollen, *O. extensa* (CNU-MEC-NN2006046). **b**, **d**, **f**, **h**, **j** Habitus, first metatarsal segments of (**a**, **c**, **e**, **g**, **i**). Scale bars represent: 4 mm in (**a**, **c**, **g**, **h**, **j**); 0.5 mm in (**b**, **d**, **e**, **g**, **i**)

**Fig. 3:**
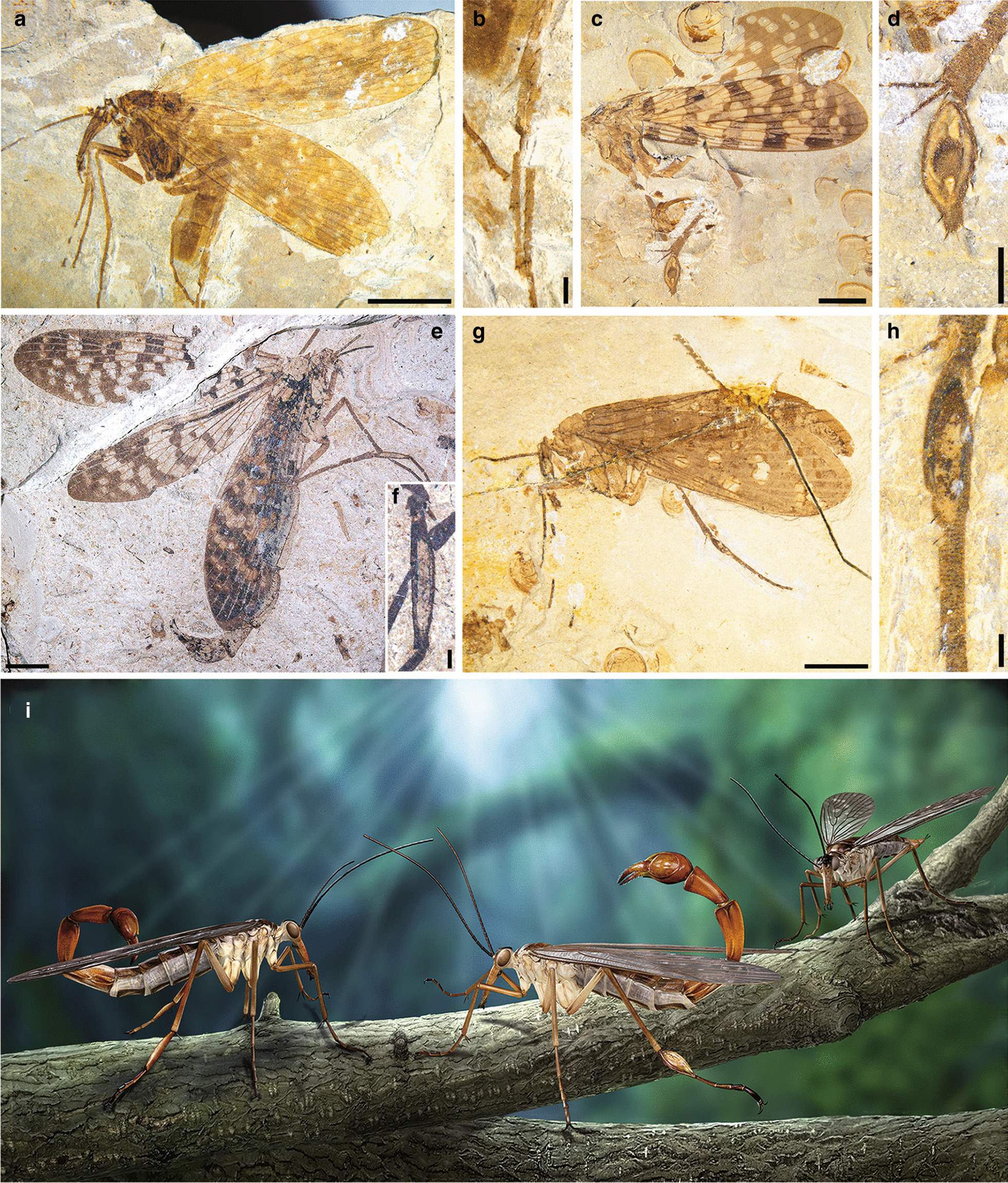
Comparison of non-swollen versus swollen first metatarsal segments. **a**, **b** Non-swollen, *Mesopanorpa densa* (CNU-MEC-NN2016270). **c**, **d** Heavily swollen, *Mesopanorpa luanpingensis* (CNU-MEC-NN2016229). **e**, **f** Slightly swollen, *Orthophlebia elenae* (CNU-MEC-NN2014020). **g**, **h** Moderately swollen, *Mesopanorpa densa* (CNU-MEC-NN2016253). **i** Artist’s reconstruction of orthophlebiids with the first metatarsal segment slightly swollen (male at left) or extremely swollen (male at center) (credit: Dr. Chen Wang). **b**, **d**, **f**, **h** Habitus, first metatarsal segments of (**a**, **c**, **e**, **g**). Scale bars represent: 4 mm for (**a**, **c**, **e**, **g**); 0.5 mm for (**b**, **d**, **f**, **h**)

We identified five discrete categories of metatarsal swelling: non-swollen (Figs. 2a, b and 3a, b), slightly swollen (Figs. 2c,  d and 3e, f), moderately swollen (Figs. 2e, f and 3g, h), heavily swollen (Figs. 2g, j; 3c, d) and extremely swollen (Fig. 2 h, i), respectively. The cuticles of the first metatarsal segments corresponding to non-swollen (Fig. 2b) or slightly swollen categories (Figs. 2d and 3f) are typically light-toned, smooth and continuous. Segments corresponding to moderately (Fig. 2e) and heavily swollen (Fig. 2g) categories typically show a light-toned central region enveloped by darker-toned margins (Fig. 3d, h). These differences in visual tone, in particular in local light-toned cuticle regions, may indicate the presence of a locally thinner cuticle as a result of cuticle swelling. Extremely swollen segments frequently exhibit dark-toned irregular lines and blotches and extensive fracturing of the cuticle, sometimes exposing the interior of the metatarsus (Fig. 2i).

### (A) Statistical analysis

**Fig. 4:**
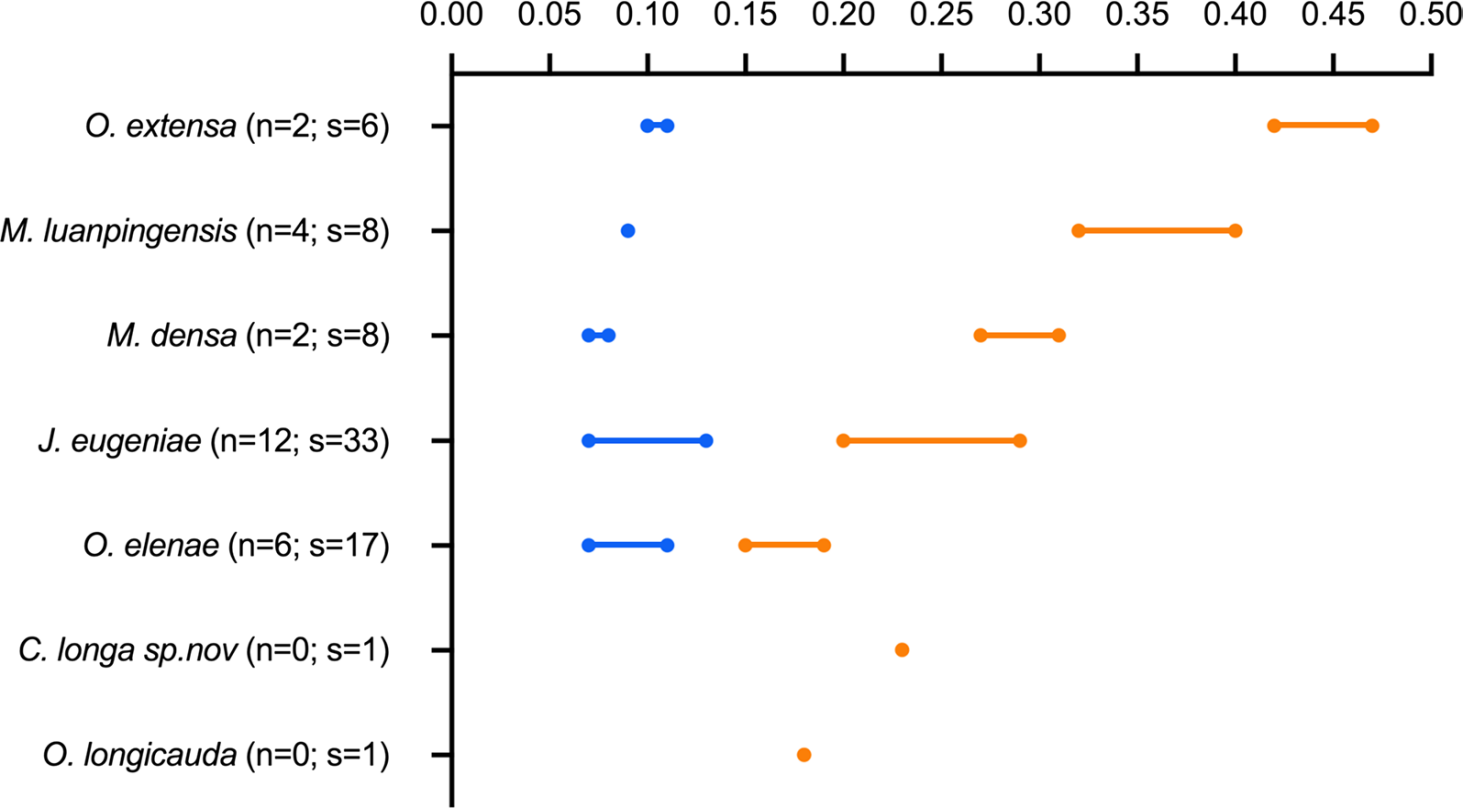
Rank order plot of the aspect ratio (AR) of each scorpionfly taxon. Note wide variations in AR of swollen tarsi relative to non-swollen ones. The orange lines denote swollen tarsi, and blue lines represent non-swollen ones. s = number of specimens having swollen tarsi; n = number of specimens having non-swollen tarsi. The data for *O. longicauda* is obtained from Willmann and Novokshonov (1998). y-axis: Species; x-axis: Aspect ratio (AR)

Our dataset includes 87 scorpionfly specimens: one holcorpid and 86 orthophlebiids represent six species belonging to four genera from the Middle Jurassic of Northeastern China (Additional file 1: Figures S1–S5). The aspect ratio (AR) of the first metatarsal segment (width/length) was used as a proxy for the degree of swelling. The AR of the metatarsus is 0.09 ± 0.02 in specimens with non-swollen metatarsi (n = 24) and 0.26 ± 0.08 in specimens with swollen ones (n = 50), respectively (We were unable to obtain measurement data for the remaining specimens due to incomplete preservation of the metatarsi). Differences between these two groups are statistically significant (two-tailed t = 10.88, p < 0.0001). For each taxon, the AR of swollen metatarsi is markedly higher than that of non-swollen metatarsi (Fig. 4) and each taxonomic group has a relatively distinct range in shape of metatarsi (Tables 1 and 2). As shown in Fig. 4, O. *elenae* (AR: 0.15–0.19, s(n) = 17) was classified as slightly swollen, *J. eugeniae* (AR: 0.20–0.29, s(n) = 33) and *M. densa* (AR: 0.27–0.31, s(n) = 8) as moderately swollen, *M. luanpingensis* (AR: 0.32–0.40,s(n) = 8) as heavily swollen, and *O. extensa* as extremely swollen (AR: 0.42–0.47, s(n) = 6). The metatarsal AR is 0.23 in one holcorpid, *Conicholcorpa longa* sp. nov., and 0.21 in one specimen of *Orthophlebia longicauda* [10]. The ARs of *J. eugeniae* (0.20–0.29) and *M. densa* (0.27–0.31) overlap, while the other three species of orthophlebiids have distinctly different aspect ratios.

**Table 1:** Measurement data for specimens with non-swollen metatarsi

ID	Taxa	Sex	Forewing length (mm)	Swollen 1st tarsal seg. length, L (mm)	Swollen 1st tarsal seg. width, W (mm)	Tibial length (mm)	Tibia Width at distal end (mm)	1st tarsal seg. ratio (W/L)	Tibia ratio (W/L)	Width ratio (1st tarsal seg./tibia	Length ratio (1st tarsal seg./tibia)	Length ratio (1st tarsal seg./forewing)	Length ratio (tibia/forewing)
2014032	*Orthophlebia elenae*	M	18.93	3.70	0.26	6.06	0.48	0.07	0.08	0.54	0.61	0.20	0.32
2014035	*Orthophlebia elenae*	U	21.18	3.14	0.31	6.82	0.50	0.10	0.07	0.62	0.46	0.15	0.32
2014043	*Orthophlebia elenae*	U		2.09	0.19	3.56	0.29	0.09	0.08	0.66	0.59		
2014060	*Orthophlebia elenae*	M	23.80	2.72	0.31			0.11				0.11	
2006033L	*Orthophlebia elenae*	M	11.80	2.30	0.21	3.48	0.40	0.09	0.11	0.53	0.66	0.19	0.29
2006033R	*Orthophlebia elenae*	M		2.08	0.21	3.50	0.38	0.10	0.11	0.55	0.59		
PI 11782	*Orthophlebia extensa*	F	23.67	3.10	0.33		0.67	0.11		0.49		0.13	
2014059	*Orthophlebia extensa*	F	19.50	3.00	0.31	7.60	0.50	0.10	0.07	0.62	0.39	0.15	0.39
2006016	*Mesopanorpa luanpingensis*	U	15.60	2.90	0.27	6.35	0.40	0.09	0.06	0.68	0.46	0.19	0.41
2006030	*Mesopanorpa luanpingensis*	U	13.70	1.94	0.18	4.14	0.35	0.09	0.08	0.51	0.47	0.14	0.30
2014051	*Mesopanorpa luanpingensis*	U	15.53	2.82	0.26	5.61	0.38	0.09	0.07	0.68	0.50	0.18	0.36
2016142	*Mesopanorpa luanpingensis*	U	19.17	3.29	0.30	5.93	0.39	0.09	0.07	0.77	0.55	0.17	0.31
2016270	*Mesopanorpa densa*	F	13.74	2.51	0.19	4.39	0.40	0.08	0.09	0.48	0.57	0.18	0.32
2016304	*Mesopanorpa densa*	U		3.90	0.29	7.80	0.46	0.07	0.06	0.63	0.50		
2016109	*Juraphlebia eugeniae*	U	17.01	3.50	0.28			0.08				0.21	
2014034	*Juraphlebia eugeniae*	U	16.72	2.65	0.21	5.26	0.46	0.08	0.09	0.46	0.50	0.16	0.31
2014038	*Juraphlebia eugeniae*	F	17.08	3.27	0.23		0.60	0.07		0.38		0.19	
2014046	*Juraphlebia eugeniae*	F	16.57	3.15	0.25	6.12	0.49	0.08	0.08	0.51	0.51	0.19	0.37
2014056	*Juraphlebia eugeniae*	U	18.65	2.41	0.18			0.07				0.13	
2014058	*Juraphlebia eugeniae*	U	17.13										
2014064	*Juraphlebia eugeniae*	F	18.90	3.43	0.29	6.62	0.73	0.08	0.11	0.40	0.52	0.18	0.35
2014065	*Juraphlebia eugeniae*	U	17.39										
2016338	*Juraphlebia eugeniae*	U	17.74	3.34	0.24	5.25	0.45	0.07	0.09	0.53	0.64	0.19	0.30
PI 11784	*Juraphlebia eugeniae*	F	18.45	3.15	0.26	5.53	0.34	0.08	0.06	0.76	0.57	0.17	0.30
PI 11783	*Juraphlebia eugeniae*	U		3.00	0.31			0.10					
2014033	*Juraphlebia eugeniae*	U	19.67	2.17	0.29	3.01	0.29	0.13	0.10	1.00	0.72	0.11	0.15

**Table 2:** Measurement data for specimens with swollen first metatarsal segments

ID	Taxa	Sex	Forewing length (mm)	Swollen 1st tarsal seg. length, L (mm)	Swollen 1st tarsal seg. width, W (mm)	Tibial length (mm)	Tibia Width at distal end (mm)	1st tarsal seg. ratio (W/L)	Tibia ratio (W/L)	Width ratio (1st tarsal seg./tibia	Length ratio (1st tarsal seg./tibia)	Length ratio (1st tarsal seg./forewing)	Length ratio (tibia/forewing)
2006028	*Orthophlebia elenae*	M	19.17	2.70	0.43	6.24	0.48	0.16	0.08	0.90	0.43	0.14	0.33
2014004	*Orthophlebia elenae*	U	19.14	3.00	0.53	6.57	0.62	0.18	0.09	0.85	0.46	0.16	0.34
2014005	*Orthophlebia elenae*	M	21.59	3.13	0.50	7.02	0.45	0.16	0.06	1.11	0.45	0.14	0.33
2014016	*Orthophlebia elenae*	M	23.78	3.04	0.58	6.89	0.61	0.19	0.09	0.95	0.44	0.13	0.29
2016134	*Orthophlebia elenae*	M	19.75	2.39	0.44			0.18				0.12	
2016212	*Orthophlebia elenae*	M	22.90	3.20	0.52	7.10	0.49	0.16	0.07	1.06	0.45	0.14	0.31
2016224	*Orthophlebia elenae*	M	25.43	3.73	0.56	7.80	0.60	0.15	0.08	0.93	0.48	0.15	0.31
2016227	*Orthophlebia elenae*	M	23.32	3.16	0.54	7.32	0.60	0.17	0.08	0.90	0.43	0.14	0.31
2016334	*Orthophlebia elenae*	M		2.64	0.42			0.16					
PI 11778	*Orthophlebia elenae*	M	18.97	2.59	0.43	6.44	0.45	0.17	0.07	0.96	0.40	0.14	0.34
PI 11779	*Orthophlebia elenae*	M	19.71	2.64	0.43		0.47	0.16		0.91		0.13	
2014020L	*Orthophlebia elenae*	M	23.25	3.27	0.56	7.24	0.63	0.17	0.09	0.89	0.45	0.14	0.31
2014020R	*Orthophlebia elenae*	M	23.25	3.29	0.57		0.68	0.17		0.84		0.14	
2016213L	*Orthophlebia elenae*	M		3.39	0.58	7.49	0.47	0.17	0.06	1.23	0.45		
2016213R	*Orthophlebia elenae*	M		3.34	0.51	7.56	0.49	0.15	0.06	1.04	0.44		
2016228L	*Orthophlebia elenae*	M			0.52								
2016228R	*Orthophlebia elenae*	M	19.98	3.11	0.50	6.45	0.59	0.16	0.09	0.85	0.48	0.16	0.32
2006046	*Orthophlebia extensa*	M	18.91	5.20	2.25		0.62	0.43		3.63		0.27	
2009622	*Orthophlebia extensa*	M	18.08	> 5.28	2.04								
2014068	*Orthophlebia extensa*	M	18.65	5.31	2.23			0.42				0.28	
2016345	*Orthophlebia extensa*	M	17.70	4.58	> 1.59							0.26	
2014022L	*Orthophlebia extensa*	U	18.17	4.13	1.90	> 2.4	0.74	0.46		2.57		0.23	
2014022R	*Orthophlebia extensa*	U		4.26	2.01	> 2.33	0.73	0.47		2.75			
2014012	*Mesopanorpa luanpingensis*	U	18.99	> 3.3	0.94								
2016158	*Mesopanorpa luanpingensis*	U		2.63	1.05			0.40					
2016222	*Mesopanorpa luanpingensis*	U	15.15	2.70	0.87	4.75	0.49	0.32	0.10	1.78	0.57	0.18	0.31
2016229	*Mesopanorpa luanpingensis*	U	16.84	> 2.51	1.00								
PI 11785	*Mesopanorpa luanpingensis*	U	19.13	2.90	1.12			0.39				0.15	
2016233	*Mesopanorpa luanpingensis*	U	18.70	> 3.36	1.08		0.67			1.61			
2016269	*Mesopanorpa luanpingensis*	U		2.65	0.84			0.32					
2016329	*Mesopanorpa luanpingensis*	U	17.35		0.90								
2016348	*Mesopanorpa luanpingensis*	U	16.10	> 3.35	0.95								
2014003	*Mesopanorpa densa*	M	25.30	3.87	1.18	4.90	0.60	0.30	0.12	1.97	0.79	0.15	0.19
2014024	*Mesopanorpa densa*	M	22.10	> 2.46	0.76	4.60	0.45		0.10	1.69			0.21
2016211	*Mesopanorpa densa*	U		3.29	1.06	5.99	0.53	0.32	0.09	2.00	0.55		
2016253	*Mesopanorpa densa*	U	17.88	3.17	> 0.51	6.58	0.47		0.07		0.48	0.18	0.37
2014010L	*Mesopanorpa densa*	U	18.33	2.70	0.82	5.26	0.61	0.30	0.12	1.34	0.51	0.15	0.29
2014010R	*Mesopanorpa densa*	U	18.33	2.74	0.97	5.19	0.60	0.35	0.12	1.62	0.53	0.15	0.28
2014023	*Mesopanorpa densa*	M	26.24	4.40	1.34	6.38	0.85	0.30	0.13	1.58	0.69	0.17	0.24
2014021	*Mesopanorpa densa*	M	23.20	4.02	1.23			0.31				0.17	
2014001	*Juraphlebia eugeniae*		18.77	3.65	1.00	6.38	0.61	0.27	0.10	1.64	0.57	0.19	0.34
2014002	*Juraphlebia eugeniae*	M	18.38	3.67	1.03	7.60	0.50	0.28	0.07	2.06	0.48	0.20	0.41
2014007	*Juraphlebia eugeniae*	M	17.76	3.80	1.00	5.74	0.48	0.26	0.08	2.08	0.66	0.21	0.32
2014008	*Juraphlebia eugeniae*	U	17.40	3.70	1.08	6.33	0.68	0.29	0.11	1.59	0.58	0.21	0.36
2014011	*Juraphlebia eugeniae*	U	15,80		0.79								
2014014	*Juraphlebia eugeniae*	U		3.57	1.05			0.29					
2014017	*Juraphlebia eugeniae*	M		3.67	1.08			0.29					
2014018	*Juraphlebia eugeniae*	U	16.05	3.39	0.85	5.44	0.56	0.25	0.10	1.52	0.62	0.21	0.34
2014025	*Juraphlebia eugeniae*	U	17.40	3.65	1.02	6.57	0.65	0.28	0.10	1.57	0.56	0.21	0.38
2014027	*Juraphlebia eugeniae*	M	18.77	3.64	0.95	6.08	0.60	0.26	0.10	1.58	0.60	0.19	0.32
2014028	*Juraphlebia eugeniae*	U	18.30	3.30	0.92		0.57	0.28		1.61		0.18	
2014054	*Juraphlebia eugeniae*	U	16.05	3.02	0.86	5.47	0.50	0.28	0.09	1.72	0.55	0.19	0.34
2016215	*Juraphlebia eugeniae*	U	17.86	3.88	0.96	5.60	0.56	0.25	0.10	1.71	0.69	0.22	0.31
2016216	*Juraphlebia eugeniae*	U	19.94	3.76	1.07	6.14	0.65	0.28	0.11	1.65	0.61	0.19	0.31
2016217	*Juraphlebia eugeniae*	U	16.02	3.23	0.86	5.75	0.58	0.27	0.10	1.48	0.56	0.20	0.36
2016218	*Juraphlebia eugeniae*	U		3.29	0.84			0.26					
2016221	*Juraphlebia eugeniae*	U	15.83	3.18	0.92	5.60	0.56	0.29	0.10	1.64	0.57	0.20	0.35
PI 11781	*Juraphlebia eugeniae*	U	16.02		1.02								
2014009L	*Juraphlebia eugeniae*	U	19.25	3.68	1.04	5.83	0.69	0.28	0.12	1.51	0.63	0.19	
2014009R	*Juraphlebia eugeniae*	U		3.51	1.01	6.00	0.65	0.29	0.11	1.55	0.59		
2014015L	*Juraphlebia eugeniae*	M	18.50	3.20	0.76	6.16		0.24	0.00		0.52	0.17	0.33
2014015R	*Juraphlebia eugeniae*	M		3.40	0.84	6.25	0.54	0.25	0.09	1.56	0.54		
2014019L	*Juraphlebia eugeniae*	M	15.77	3.20	0.89	5.19	0.52	0.28	0.10	1.71	0.62	0.20	0.33
2014019R	*Juraphlebia eugeniae*	M		3.09	0.80	5.25	0.50	0.26	0.10	1.60	0.59		
2014026L	*Juraphlebia eugeniae*	U	17.80	3.21	0.86	5.60		0.27	0.00		0.57	0.18	0.31
2014026R	*Juraphlebia eugeniae*	U		3.40	0.83	5.40	0.54	0.24	0.10	1.54	0.63		
2014029L	*Juraphlebia eugeniae*	U	18.16	3.65	0.72	5.30	0.45	0.20	0.08	1.60	0.69	0.20	0.29
2014029R	*Juraphlebia eugeniae*	U		3.76	0.82			0.22					
2014030L	*Juraphlebia eugeniae*	U	15.85	3.21	0.91	6.01		0.28	0.00		0.53	0.20	0.38
2014030R	*Juraphlebia eugeniae*	U		3.00	0.88	6.17	0.55	0.29	0.09	1.60	0.49		
2015116L	*Juraphlebia eugeniae*	M	18.16	3.50	0.98	6.13	0.60	0.28	0.10	1.63	0.57	0.19	0.34
2015116R	*Juraphlebia eugeniae*	M		3.69	0.96	6.13	0.64	0.26	0.10	1.50	0.60		
PI 11780	*Juraphlebia eugeniae*	M	16.24	> 2.93	1.88	5.57	0.44		0.08	4.27			0.34
2015108	*Conicholcorpa longa sp. nov*	M	20.18	3.00	0.70	6.64	0.57	0.23	0.09	1.23	0.45	0.15	0.33

Measurement data (Tables 1 and 2) for specimens (with non-swollen or swollen first metatarsal segments) of five species of Orthophlebiidae and one of Holcorpidae were analysed as follows:

*The AR of the first metatarsal segment* Specimens lacking measurable data were excluded. The mean and standard deviation for AR of non-swollen examples (n = 24) of the first metatarsal segment is 0.09 ± 0.02, and those for swollen examples (n = 50) is 0.26 ± 0.08. The t-test result (t = 10.88, df = 83, p < 0.0001) indicates that these two groups are statistically different. The higher standard deviation values for the swollen metatarsal segments indicate greater variability in swelling morphology than the non-swollen counterparts.

*The length ratios of 1st metatarsal segment/metatibia* We also calculated the ratios of the length of the first metatarsal segment to that of the metatibia. The mean and standard deviation for non-swollen (n = 18) and swollen (n = 45) specimens are 0.55 ± 0.08 and 0.55 ± 0.09, respectively. The t-test result (t = 0.002, df = 61, p = 0.98) demonstrates that these two groups are statistically similar, suggesting that swelling did not affect the length of the 1st metatarsal segment.

*The AR of the 1st metatarsal segments for non‐swollen males/non-swollen females* We calculated the AR’s of the first metatarsal segment for non-swollen and swollen representatives of males and females. The mean and standard deviation of ARs for non-swollen tarsi of males (n = 4) and females (n = 7) are 0.09 ± 0.02 and 0.09 ± 0.01, respectively. The t-test result demonstrates that the differences in non-swollen tarsi between males and females are not statistically significant. The AR’s cannot be compared for swollen tarsi between males (n = 31) and females (n = 0), due to lack of swollen tarsi in female specimens.

### (B) Geometric morphometric analyses (GMA)

We conducted GMA for the same set of 87 scorpionfly specimens as mentioned above. The metatarsi show the non-swollen condition (i.e., the margins of the metatarsi are oriented approximately parallelly to each other) in 25 specimens (Figs. 2a, b and 3a, b). Sixty-one orthophlebiids show swollen first metatarsal segments (Fig. 2d, e, g, i). Sex could not be determined for 32 of these specimens due to poor preservation; all of the remaining 29 specimens are males.

**Fig. 5:**
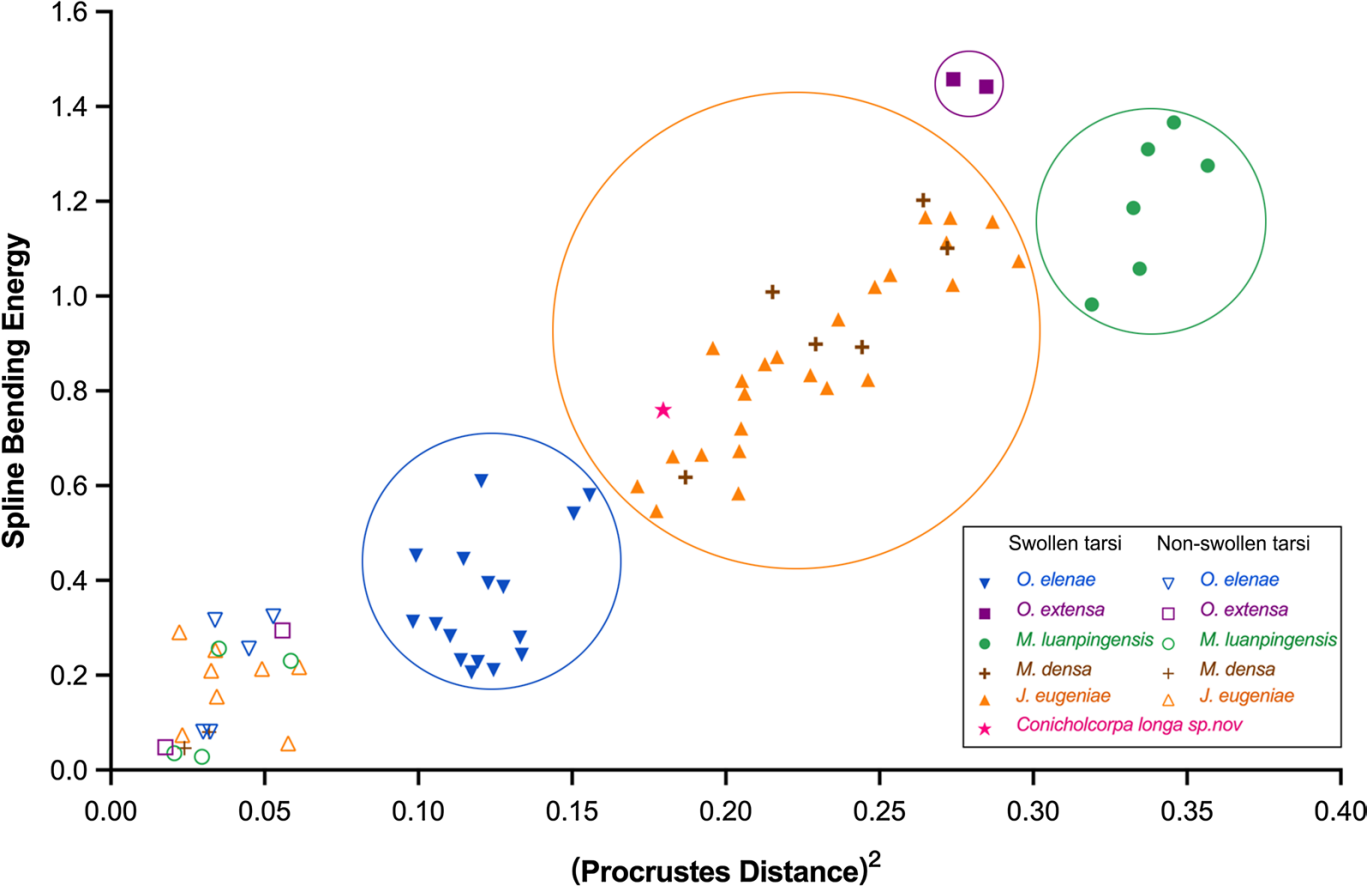
Geometric morphometric analyses (GMA) of the first metatarsal segments in non-swollen and swollen morphotypes. Swollen metatarsi have higher and more variable values for both (Procrustes distance)^2^ and spline bending energy, and each taxon group has a relatively certain range

Calculations of the Procrustes distance and Spline bending energy for each specimen show that non-swollen metatarsi have consistently low Procrustes distance and Spline bending energy (Fig. 5). In contrast, swollen metatarsi have higher and more variable values for both Procrustes distance and spline bending energy, reflecting more distortion and more variable first metatarsal geometries. Furthermore, the results of GMA also show that three species, *O. extensa, O. elenae* and *M. luanpingensis* with swollen metatarsi have distinctly different data ranges. However, *M. densa* and *J. eugeniae* have overlapping data ranges, consistent with the AR data in Fig. 4.

## Discussion

### Metatarsal swellings in diverse scorpionflies

In this study, swollen first metatarsal segments have been reported in two families, four genera and seven species of Panorpoidea. Discovery of numerous specimens that show this feature indicates that swollen metatarsal first segments were relatively common in Orthophlebiidae in the mid-Mesozoic.

### The species‐specific of metatarsal swelling in scorpionflies

Our data show that the shape of the swollen metatarsi is diverse in orthophlebiids and is distinct at the species level. These morphological characters appear to be taxonomically and phylogenetically informative.

Based on measurement data for specimens with non-swollen or swollen first metatarsal segments as shown in Tables 1 and 2, statistical analyses as shown in Fig. 4 and GMA of the first metatarsal segments in non-swollen and swollen morphotypes as shown in Fig. 5; Table 3, we discovered that all fossil specimens with swollen first metatarsal segments are males in contrast to the females and three male specimens of *O. elenae* having non-swollen tarsi. We suggest that swollen metatarsi might be related to sexual dimorphism. The GMA results in Fig. 5 clearly indicate the level of swelling for four species of orthophlebiids, i.e., *O. extensa, M. luanpingensis, M. densa* and *J. eugeniae*, not having males with non-swollen first metatarsal segments, is much higher and more significant than the swelling of male *O. elenae*.

**Table 3:** Summary of relative Procrustes distance and spline bending energy for non-swollen and swollen specimens

Metatarsal 1st segment	Specimen ID	Species	(Procrustes distance)^2^		Spline bending energy
Non-swollen	2014032	*Orthophlebia elenae*	0.04494	0.25617	
Non-swollen	2014035	*Orthophlebia elenae*	0.03385	0.316470	
Non-swollen	2014043	*Orthophlebia elenae*	0.03238	0.08051	
Non-swollen	2014060	*Orthophlebia elenae*	0.052790	0.324300	
Non-swollen	2006033L	*Orthophlebia elenae*	0.02668	0.0826	
Non-swollen	2006033R	*Orthophlebia elenae*	0.03338	0.07957	
Non-swollen	PI 11782	*Orthophlebia extensa*	0.01777	0.04785	
Non-swollen	2014059	*Orthophlebia extensa*	0.0558	0.29472	
Non-swollen	2006016	*Mesopanorpa luanpingensis*	0.03513	0.25596	
Non-swollen	2006030	*Mesopanorpa luanpingensis*	0.0585	0.2302	
Non-swollen	2014051	*Mesopanorpa luanpingensis*	0.02061	0.03524	
Non-swollen	2016142	*Mesopanorpa luanpingensis*	0.02958	0.02774	
Non-swollen	2016270	*Mesopanorpa densa*	0.03192	0.08019	
Non-swollen	2016304	*Mesopanorpa densa*	0.02396	0.04617	
Non-swollen	2016109	*Juraphlebia eugeniae*	0.05763	0.05643	
Non-swollen	2014034	*Juraphlebia eugeniae*	0.04916	0.2134	
Non-swollen	2014038	*Juraphlebia eugeniae*	0	0	
Non-swollen	2014046	*Juraphlebia eugeniae*	0.0223	0.39066	
Non-swollen	2014056	*Juraphlebia eugeniae*	0.03261	0.20964	
Non-swollen	2014064	*Juraphlebia eugeniae*	0.03447	0.15473	
Non-swollen	2016338	*Juraphlebia eugeniae*	0.02325	0.07408	
Non-swollen	PI 11783	*Juraphlebia eugeniae*	0.06125	0.21697	
Non-swollen	PI 11784	*Juraphlebia eugeniae*	0.03397	0.25296	
Swollen	2006028	*Orthophlebia elenae*	0.11472	0.44573	
Swollen	2014004	*Orthophlebia elenae*	0.15569	0.58	
Swollen	2014005	*Orthophlebia elenae*	0.09829	0.31304	
Swollen	2014016	*Orthophlebia elenae*	0.15055	0.54123	
Swollen	2016134	*Orthophlebia elenae*	0.09923	0.45225	
Swollen	2016212	*Orthophlebia elenae*	0.11929	0.22794	
Swollen	2016224	*Orthophlebia elenae*	0.11383	0.23143	
Swollen	2016227	*Orthophlebia elenae*	0.12271	0.39442	
Swollen	2016334	*Orthophlebia elenae*	0.10575	0.30799	
Swollen	PI 11778	*Orthophlebia elenae*	0.12446	0.21122	
Swollen	PI 11779	*Orthophlebia elenae*	0.13363	0.24293	
Swollen	2014020L	*Orthophlebia elenae*	0.12047	0.60943	
Swollen	2014020R	*Orthophlebia elenae*	0.11034	0.2826	
Swollen	2016213L	*Orthophlebia elenae*	0.11444	0.25325	
Swollen	2016213R	*Orthophlebia elenae*	0.15175	0.3055	
Swollen	2016228L	*Orthophlebia elenae*	0.10943	0.17148	
Swollen	2016228R	*Orthophlebia elenae*	0.12513	0.24025	
Swollen	2016232	*Orthophlebia elenae*	0.12764	0.38622	
Swollen	2006046	*Orthophlebia extensa*	0.28473	1.44194	
Swollen	20096222	*Orthophlebia extensa*	0.27395	1.45784	
Swollen	2016158	*Mesopanorpa luanpingensis*	0.35679	1.27539	
Swollen	2016329	*Mesopanorpa luanpingensis*	0.34577	1.3663	
Swollen	2016222	*Mesopanorpa luanpingensis*	0.31902	0.9822	
Swollen	2016229	*Mesopanorpa luanpingensis*	0.33734	1.30988	
Swollen	PI 11785	*Mesopanorpa luanpingensis*	0.33459	1.05804	
Swollen	2016233	*Mesopanorpa luanpingensis*	0.33253	1.1858	
Swollen	2014003	*Mesopanorpa densa*	0.22926	0.89847	
Swollen	2014010L	*Mesopanorpa densa*	0.26062	1.14765	
Swollen	2014010R	*Mesopanorpa densa*	0.34781	1.65662	
Swollen	2014021	*Mesopanorpa densa*	0.18693	0.61767	
Swollen	2014023	*Mesopanorpa densa*	0.24431	0.8924	
Swollen	2016221	*Mesopanorpa densa*	0.21518	1.00841	
Swollen	2014024	*Mesopanorpa densa*	0.27208	1.10139	
Swollen	2014001	*Juraphlebia eugeniae*	0.19577	0.89062	
Swollen	2014002	*Juraphlebia eugeniae*	0.21672	0.87158	
Swollen	2014007	*Juraphlebia eugeniae*	0.25346	1.04426	
Swollen	2014008	*Juraphlebia eugeniae*	0.23662	0.95072	
Swollen	2014014	*Juraphlebia eugeniae*	0.2738	1.02411	
Swollen	2014017	*Juraphlebia eugeniae*	0.22751	0.83356	
Swollen	2014018	*Juraphlebia eugeniae*	0.17129	0.59885	
Swollen	2014025	*Juraphlebia eugeniae*	0.19211	0.66528	
Swollen	2014027	*Juraphlebia eugeniae*	0.28677	1.15687	
Swollen	2014054	*Juraphlebia eugeniae*	0.29526	1.07421	
Swollen	2014028	*Juraphlebia eugeniae*	0.26493	1.16629	
Swollen	2016215	*Juraphlebia eugeniae*	0.20442	0.67271	
Swollen	2016216	*Juraphlebia eugeniae*	0.23296	0.80632	
Swollen	2016217	*Juraphlebia eugeniae*	0.20412	0.58381	
Swollen	2016211	*Juraphlebia eugeniae*	0.24622	0.823	
Swollen	2014009L	*Juraphlebia eugeniae*	0.24013	0.97214	
Swollen	2014009R	*Juraphlebia eugeniae*	0.25676	1.06644	
Swollen	2014015L	*Juraphlebia eugeniae*	0.17079	0.63429	
Swollen	2014015R	*Juraphlebia eugeniae*	0.19465	0.68952	
Swollen	2014019L	*Juraphlebia eugeniae*	0.19402	0.79919	
Swollen	2014019R	*Juraphlebia eugeniae*	0.23133	0.91442	
Swollen	2014026L	*Juraphlebia eugeniae*	0.1952	0.79003	
Swollen	2014026R	*Juraphlebia eugeniae*	0.21481	0.65204	
Swollen	2014029L	*Juraphlebia eugeniae*	0.18162	0.68703	
Swollen	2014029R	*Juraphlebia eugeniae*	0.13321	0.40661	
Swollen	2014030L	*Juraphlebia eugeniae*	0.28065	1.16283	
Swollen	2014030R	*Juraphlebia eugeniae*	0.26533	1.16815	
Swollen	2015116L	*Juraphlebia eugeniae*	0.21063	0.82531	
Swollen	2015116R	*Juraphlebia eugeniae*	0.20159	0.76289	
Swollen	PI 11780	*Juraphlebia eugeniae*	0.27179	1.11337	
Swollen	PI 11781	*Juraphlebia eugeniae*	0.2053	0.82166	
Swollen	2015108	*Conicholcorpa longa sp. nov*	0.17963	0.75963	

These swellings were previously interpreted first as non-pathological structures used for releasing pheromones, detecting vibration or sound, grasping female mates or providing mating gifts [10], and later as “metatarsal organ[s] of unknown function” [11]. The results of our statistical analyses indicate that the extent of swelling is independent of the lengths of tibia and wing (Additional file 1: Figure S6). It is more likely to represent a specialised structure with certain functions for a specific individual, not as a “metatarsal organ” proposed by Willmann and Novokshonov [11].

### Phylogenetic relationships between Holcorpidae and Orthophlebiidae

Orthophlebiidae and Holcorpidae are two extinct families of Mecoptera established by Handlirsch and Willmann [17, 18]. The phylogenetic relationship between these two families has been controversially discussed. Willmann proposed that *Holcorpa* may be basal to the clade Panorpidae + Panorpodidae, in a lineage perhaps descended from the Mesozoic “Orthophlebiidae” [18, 19]. Grimaldi and Engel placed *Holcorpa* as a sister group to Panorpidae + Panorpodidae + Bittacidae [20]. Archibald supported that Holcorpidae arose from the orthophlebiid grade and might be a sister group to Panorpidae + Panorpodidae [21]. Orthophlebiidae is regarded to be a paraphyletic stem group of the Panorpoidea by Archibald et al. [22]. Soszyńska-Maj et al. suggested that Panorpidae and Panorpodidae do not represent the sister taxa, and Panorpidae and Orthophlebiidae form one clade [23]. The conclusion agrees with the two molecular studies on Mecoptera [24, 25]. Based on our analyses, Holcorpidae may be a sister group to Orthophlebiidae. The tarsal swelling phenomenon should be a specialization of this clade rather than a specific monophyletic group of *O. longicauda* and *O. elenae* excluding *Holcorpa* from their proposed clade in Willmann and Novokshonov [11]. Soszyńska-Maj et al. proposed that Orthophlebiidae and Panorpidae derived from common evolutionary lines, different from Panorpodidae and Protorthophlebiidae [23]. Therefore, Holcorpidae and Orthophlebiidae together with Panorpidae might have descended from a common ancestor.

### Functional suggestion about swollen metatarsal segments

Specialised structures of the legs, especially femura and tibiae, are always associated with special functions for insects, such as bees’ pollen-carrying legs, some beetle’s digging legs, mantises’ grasping legs, etc. The pollen basket is localised on the flattened and enlarged tibia of hind legs and is found in some eusocial bees [26]. Praying mantises have rows of spines on the edges of the ventral surface of the forefemur and foretibia [27]. In terms of our specimens, though some setae are found on the swollen basitarsi of scorpionflies (Additional file 1: Figure S4h), they do not seem to be used for predation. Compared with other extant insects, the swollen basitarsi are similar to the dance flies (Empididae of Diptera) with glandular cells in the basitarsi [16]. In addition, some silk production structures in webspinners are located on the forelegs [2] without any exaggerated swellings on the hind legs. However, we cannot find similar opening structures as those of dance flies or webspinners in the first swollen tarsal segment, nor detect the position of the pheromone receptor.

The enlarged structures are all located in the first metatarsal segments of the male specimens. That is why we agree with the previous view that this is a sexually dimorphic feature. Although holcorpids and orthophlebiids have extremely exaggerated male genitalia, the swollen metatarsal segments are likely to serve as an alternative supporting tool to attract potential mating partners. It is well known that extant scorpionflies (Panorpidae and Bittacidae) adopt a mating strategy by providing nuptial gifts to potential mating partners before mating or courtship to increase the likelihood of mating [28]. Orthophlebiidae and Panorpidae share a common origin [23], thus, orthophlebiids might also have the same behaviours of offering nuptial gifts. *Panorpa liui* offers only prey items rather than salivary secretions as nuptial gift, which is considered to be relatively basal [29]. Male bittacids carry nuptial gifts impaled on the beak or gripped with the hind tarsi while they hang from a perch with their front legs [30]. Orthophlebiids were more likely to carry out the nuptial gifts in a less sophisticated way. Dance flies are also unique in their mating behaviour. Males may successfully ‘cheat’ the female with inanimate objects such as a willow seed or an empty silk ‘balloon’ [31, 32]. In the study of dance flies, the size and the ornamentation of the modified fore tarsi of males of *Empis jaschhoforum*, Daugeron 2011 could play an important role in mate selection [9]. Therefore, we suggest that the function of the swollen first metatarsal segments in holcorpids and orthophlebiids have been associated with nuptial gift behaviour in order to guard the prey and disguise it as a bigger and richer gift waiting for females. This trick might have allowed the orthophlebiids to flourish in the Mid-Mesozoic, but the evolution of such functional extremes may have reduced fitness, resulting in the extinction and extinction of holcorpids and orthophlebiids later.

## Conclusions

A new species of the Holcorpidae, *Conicholcorpa longa* Zhang, Shih & Ren sp. nov., represented by a male specimen from the Middle Jurassic Jiulongshan Formation of China, has the swollen first metatarsal segments. In addition, analyses of 86 orthophlebiid fossil specimens indicate that the degree of tarsal swelling within the Orthophlebiidae species possessing swollen first metatarsal segments is species-specific. This report not only adds to the increase of the known diversity of the Mid-Mesozoic scorpionflies, but also enhances our understanding of the relationships between Orthophlebiidae and Holcorpidae, as well as implications for the extant groups including Panorpidae and Panorpodidae in the superfamily Panorpoidea. The tarsal swellings are considered to be sexual dimorphic, potentially associated with sexual display by males and/or camouflage of a “nuptial gift” in the mating process.

## Materials and methods

### Fossil specimens

All fossil specimens used in this study are from Daohugou village, Shantou Township, Ningcheng County, Inner Mongolia, Northeastern China. The Daohugou locality is considered to be one of the most important fossil insect sites globally, where a diverse insect fauna has been studied extensively [14, 33, 34].

Eight of the scorpionfly specimens used in this study are housed in the collections of the Inner Mongolia Museum of Natural History (IMMNH), Huhhot, Inner Mongolia, China (Additional file 1). All remaining specimens are categorized by Taiping Gao and housed in the fossil insect collection of the Key Laboratory of Insect Evolution and Environmental Changes, College of Life Sciences and Academy for Multidisciplinary Studies, Capital Normal University, Beijing, China. (CNUB; Dong Ren, Curator). The specimen identification consists of year and number. The environment of the Fossil Museum requires dryness, avoiding abrasion and overtaking.

### Photography and light microscopy

The specimens were examined and studied with the use of a Leica M205C stereomicroscope and photographed using a Nikon SMZ 25 stereomicroscope coupled to a Nikon DS-Ri 2 digital camera system under reflected light. Alcohol wetting was used for some specimens to enhance character observation. Line drawings were manually prepared using Adobe Illustrator CC 2020 and Adobe Photoshop CC 2020 graphics software.

### Statistical analysis

We measured all specimens and list the data in Tables 1 and 2. The aspect ratio (AR) of the first metatarsal segment (width/length) was used as a proxy for the degree of swelling.

### Geometric morphometric analyses (GMA)

GMA are commonly applied to the study of phenetic relationships among extant and fossil insects and their associations with plants [35–38]. Common parameters used in GMA include, e.g., Procrustes distance and Spline bending energy. The Procrustes distance is a measure of the difference between two outlines after the two have been ‘superimposed’ via translation, scaling and rotation, and is defined as the square root of the sum of 100 sets of the square of the distance between each specific landmark divided by the number of data points. The Spline bending energy is the energy required to bend a sample shape on a thin plate to conform to the reference shape. These indices measure similarity between normalised shapes with homologous landmarks, and the energy required to bend the shape onto a theoretical flat plate.

**Fig. 6:**
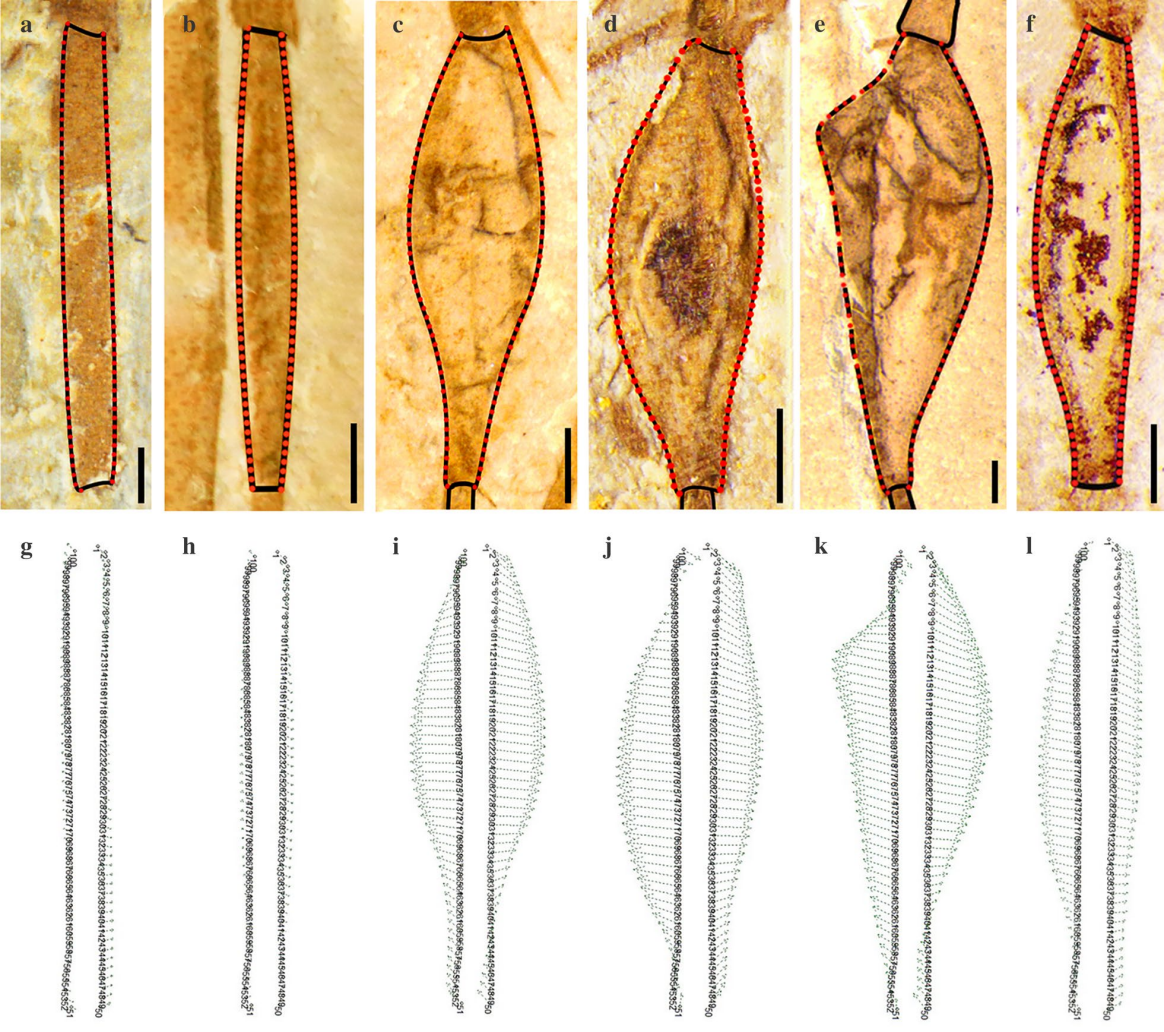
Landmark points for geometric morphometric analyses and graphic representation of geometric morphometric methods. **a**–**f** The first metatarsal segment is represented by two curves, each comprising 50 landmark points. **g**–**l** Arrows indicate Procrustes distance and Spline bending energy relative to the reference specimen, *Juraphlebia eugeniae* (CNU-MEC-NN2014038). **a**, **g** Non-swollen, *Orthophlebia extensa* (CNU-MEC-NN2014059). **b**, **h** Slightly swollen, *O. elenae* (CNU-MEC-NN2014005). **c**, **i** Moderately swollen, *Juraphlebia eugeniae* (CNU-MEC-NN2014009R). **d**, **j** Heavily swollen, *Mesopanorpa luanpingensis* (CNU-MEC-NN2016222). **e**, **k** Extremely swollen, *O. extensa* (CNU-MEC-NN2006046). **f**, **l** Slightly swollen, the new taxon of *Conicholcorpa longa* Zhang, Shih & Ren sp. nov. (CNU-MEC-NN2015108)

Digital line drawings were prepared from photographs of all scorpionflies specimens (22 non-swollen and 54 swollen) with complete first metatarsal segments (23 non-swollen and 65 swollen segments, due to inclusion of both left and right legs of one specimen having non-swollen and 11 specimens having swollen segments) and inputted into the tps-UTILS software feature [39]. The first metatarsal segment was defined by two curves (one for each of the left and right sides), each comprising 50 data points (Fig. 6), using tps-DIG; these curves were then converted into landmarks using tps-UTILS [39]. The average of all swollen and non-swollen outlines was calculated using tps-SPLIN and a global least square (GLS) reference [40]. This average was used to calculate the relative Procrustes distance and relative Spline bending energy of the dataset by comparing 100 landmarks of the studied specimens with those of a reference specimen with a non-swollen first metatarsal segment (i.e., CNU-MEC-NN2014038) (Fig. 6; Table 3). Finally, data for Spline bending energy were plotted against the square of the Procrustes distance. For background information, theory, applications, and details about the use of tps-UTILS and tps-SPLIN to model and visualize deformation, please see Bookstein and Wahba [41, 42].

## Supplementary information

**Additional file 1.** Additional Figures S1–S6.

## Data Availability

All data generated or analysed during this study are included in this published article and (its additional information files).

## Abbreviations

GMA: Geometric morphometric analyses
s/s(n): Number of swollen specimens
n: Number of specimens or number of non-swollen specimens
AR: Aspect ratio
ID: Identification
M: Male
F: Female
U: Unknown sex
Seg.: Segment
Sc: Subcostal
R_1_: First branch of the radius
Rs: Radial sector
MA: Anterior media
MP: Posterior media
CuA: Anterior cubitus
CuP: Posterior cubitus
A_1_, A_2_, A_3_: The first to third branch of the anal vein
