# Supplementary material for: Jurassic scorpionflies (Mecoptera) with swollen first metatarsal segments suggesting sexual dimorphism
Source: BMC Ecol Evol. 2021 Mar 20;21:47. doi: 10.1186/s12862-021-01771-3 (PMC7981964; doi:10.1186/s12862-021-01771-3)

**Additional file 1.**

**Figure S1-S6.** **Additional figures**

**Fig. S1** Photographs and line drawings of *Orthophlebia extensa*. New materials. **a** Habitus of a male (CNU-MEC-NN2016345); **b**, **c** forewing of (a); **d** the extremely swollen first segment of the metatarsus of (a). **e** Habitus of a male (CNU-MEC-NN2006046); **f**, **g** forewing of (e); **h** the extremely swollen first segment of the metatarsus. **i** Habitus of a female (CNU-MEC-NN2014059); **k**, **l** forewing of (i); **j** the non-swollen first segment of the metatarsus. Scale bars represent: 4 mm in (a, e, i); 2 mm in (b, c, f, g, l, l); 1 mm in (d, h, j).


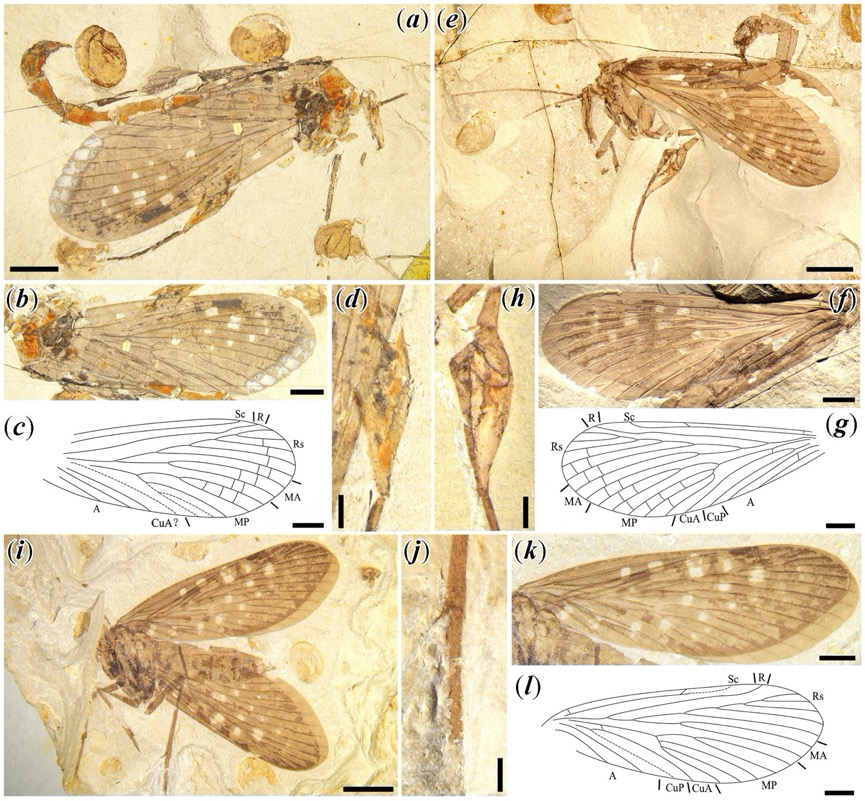


**Fig. S2** Photographs and line drawings of *Orthophlebia elenae*. New materials. **a** Habitus of an individual of unknown sex (CNU-MEC-NN2014004); **c**, **d** forewing of (a); **b** the slightly swollen first segment of the metatarsus of (a). **e** Habitus of a male (CNU-MEC-NN2014020); **f** the slightly swollen first segment of the metatarsus; **g**, **h** hind wing of (e). **l** Habitus of CNU-MEC-NN2014060, male; **i**, **j** hind wing of (l); **k** the non-swollen first segment of the metatarsus. Scale bars represent: 4 mm in (a, e, l); 2 mm in (c, d, g, h, i, j); 1 mm in (b, f, k).


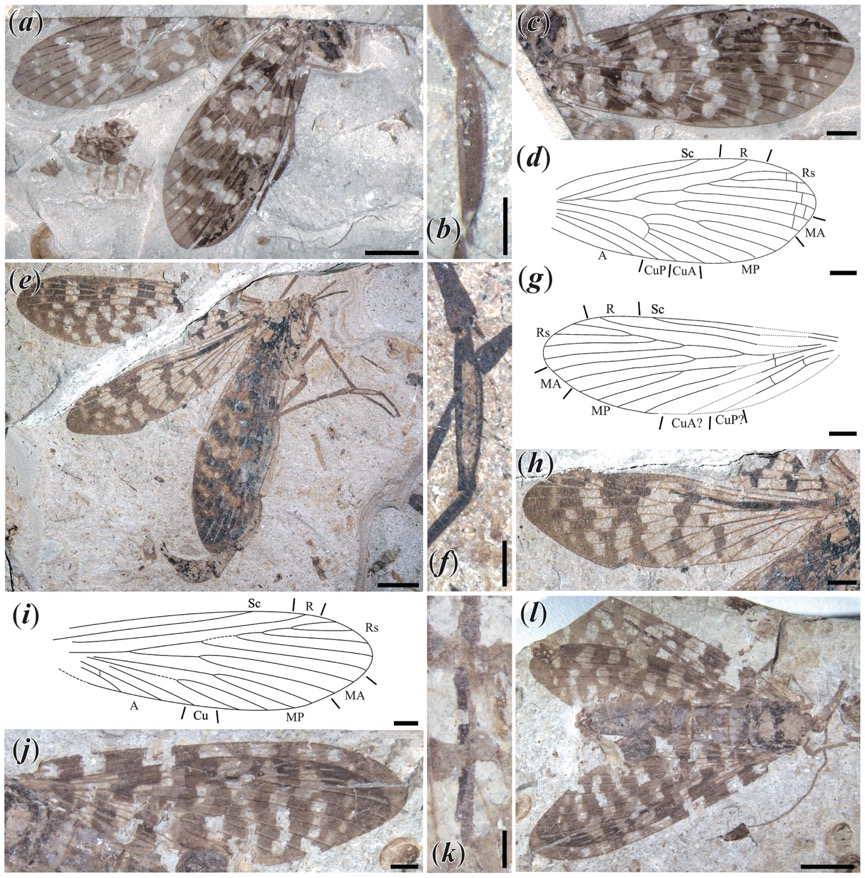


**Fig. S3** Photographs and line drawings of *Mesopanorpa densa*. New materials. **a** Habitus of male (CNU-MEC-NN2014024); **b**, **c** forewing of (a); **d** the heavily swollen first segment of the metatarsus of (a). **e** Habitus of an individual of unknown sex (CNU-MEC-NN2016253); **f**, **g** hind wing of (e); **h** the moderately swollen first segment of the metatarsus. **l** Habitus of female (CNU-MEC-NN2016270); **i**, **j** forewing of (l); **k** the non-swollen first segment of the metatarsus. Scale bars represent: 4 mm in (a, e, l); 2 mm in (b, c, f, g, i, j); 1 mm in (d, h, k).

**
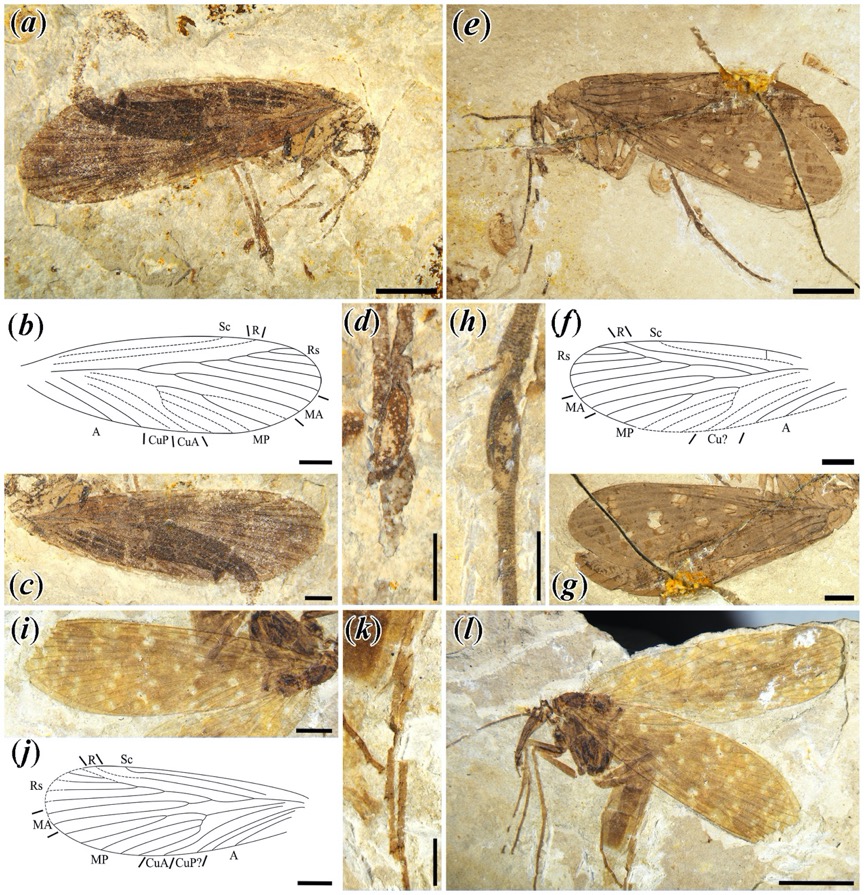
**

**Fig. S4** Photographs and line drawings of *Mesopanorpa luanpingensis*. New materials. **a** Habitus of an individual of unknown sex (CNU-MEC-NN2006016); **c**, **d** forewing of (a); **b** the non-swollen first segment of the metatarsus of (a). **g** Habitus of an individual of unknown sex (CNU-MEC-NN2016222); **e**, **f** forewing of (g); **h** the heavily swollen first segment of the metatarsus. **l** Habitus of an individual of unknown sex (CNU-MEC-NN2016229); **i**, **j** hind wing of (k); **k** the heavily swollen first segment of the metatarsus. Scale bars represent: 4 mm in (a, g, l); 2 mm in (c, d, e, f, i, j); 1 mm in (b, h, k).


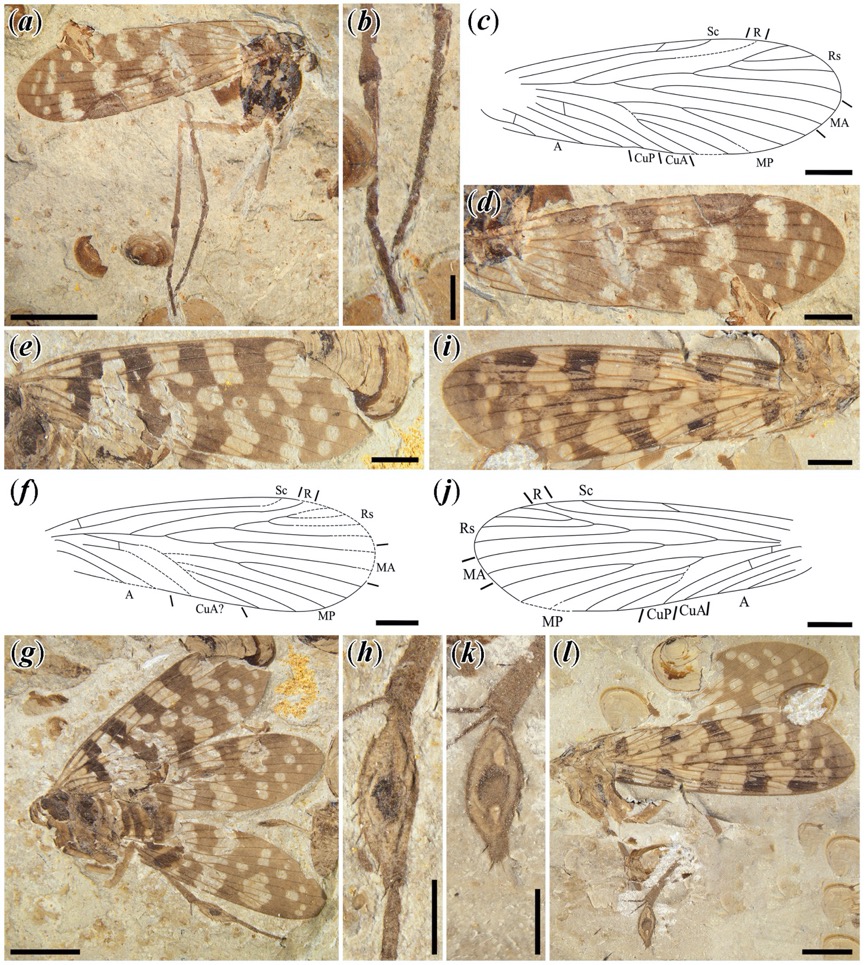


**Fig. S5** Photographs and line drawings of *Juraphlebia eugeniae*. New materials. **d** Habitus of an individual of unknown sex (CNU-MEC-NN2014033); **a**, **b** forewing of (d); **c** the non-swollen first segment of the metatarsus of (d). **e** Habitus of an individual of unknown sex (CNU-MEC-NN2014028); **g**, **h** forewing of (e); **f** the moderately swollen first segment of the metatarsus. **l** Habitus of an individual of unknown sex (CNU-MEC-NN2014001); **i**, **j** forewing of (l); **k** the moderately swollen first segment of the metatarsus. Scale bars represent: 4 mm in (d, e, l); 2 mm in (a, b, g, h, i, j); 1 mm in (c, f, k).


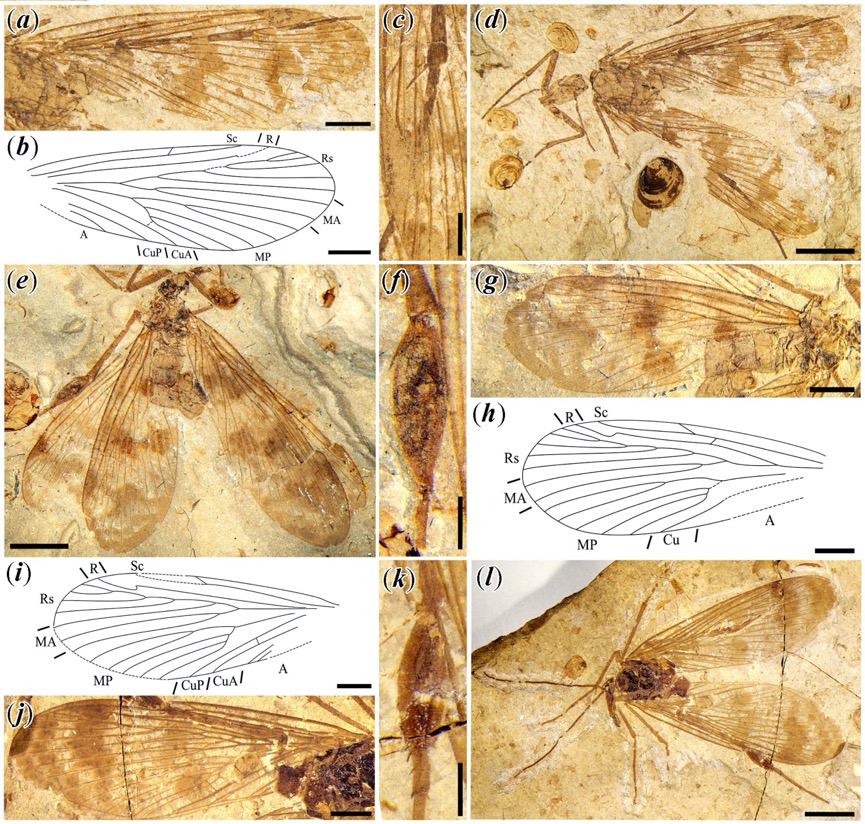


**Fig. S6** The aspect ratio of the first metatarsal segment for specimens having non-swollen and swollen tarsi versus wing length (which is used here as a proxy for body size). Trendlines are shown in dotted lines, R^2^ for non-swollen specimens is 0.187 and for swollen specimens, 0.117.


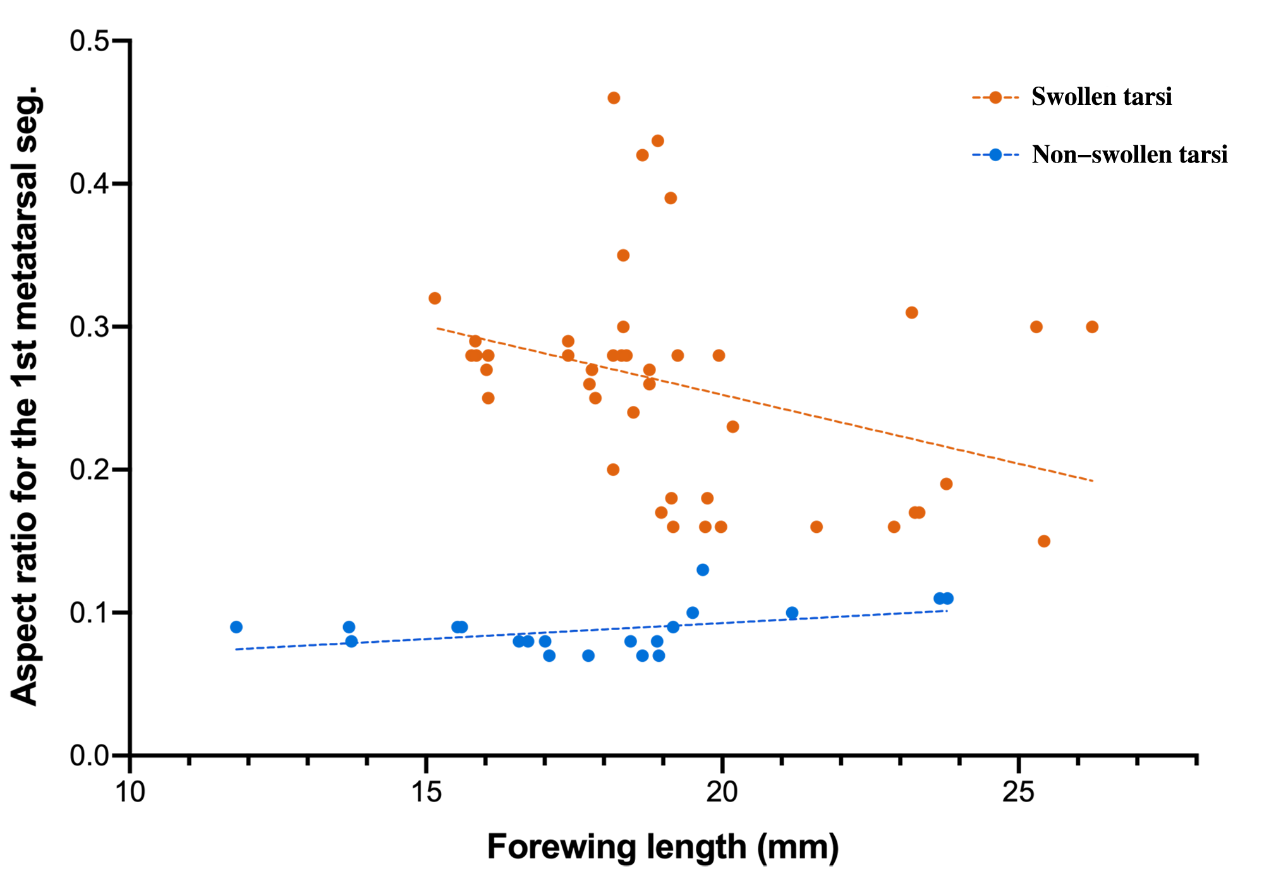

Supplement: Supplementary file 1 — Additional file 1. Additional Figures S1–S6. [file 12862_2021_1771_MOESM1_ESM.docx]
